# Practical Teaching of Business English Majors Based on Intelligent Machine Teaching

**DOI:** 10.1155/2022/3155292

**Published:** 2022-05-10

**Authors:** Yu Shi, Hu Shi

**Affiliations:** ^1^School of Foreign Languages, Shaoyang University, Shaoyang 422000, China; ^2^Software College, Dalian Jiaotong University, Dalian 116028, China

## Abstract

In order to improve the effect of business English teaching, this paper combines intelligent digital methods to intelligently process images of online business English teaching. Under the condition of weak feedback, the self-mixing interference signal has a sinusoidal shape, and the calculated envelope phase jump is small. Moreover, under moderate feedback conditions, the self-mixing interference signal is in the form of a sawtooth wave. The principle based on phase unwrapping is adopted, which solves the situation of finding the displacement very well. The experimental research shows that the practical teaching system for business English majors based on intelligent machine teaching proposed in this paper has good effects and can play an important role in business English teaching.

## 1. Introduction

Artificial intelligence is an extension of human intelligence. In the era of the explosion of information technology, the society has put forward an urgent demand for artificial intelligence, and the form of education has been reshaped. Researchers have been continuously exploring it for many years, and new technologies such as the Internet and big data have also greatly promoted the in-depth development of artificial intelligence. At present, the development of artificial intelligence has entered a new, more advanced, and more mature stage, which not only enables deep learning and cross-border integration but also has excellent characteristics such as human-machine collaboration and autonomous control [1]. Artificial intelligence technology has a good international influence, but there are still gaps between countries. Artificial intelligence courses refer to courses that are supported by artificial intelligence technology and conduct research on its principles, methods, and applications [2]. At present, the scope of research in the field of artificial intelligence continues to expand, not only in common robotics, image, and language recognition technologies but also in natural language processing and expert systems. The earliest majors were set up in the higher education stage, involving knowledge of artificial intelligence concepts, principles, technologies, and machine learning techniques and methods. In addition, with the development of society, the scope of artificial intelligence research will gradually expand [3].

In this study, because the data were collected in the real classroom environment, there are large differences between different classrooms. For example, there are inconsistencies in the classroom environment, the size of the classroom, and the placement of desks in the classroom. The difference between the obtained samples is large, and this difference is not the difference we want, and it will increase the difficulty of training. Therefore, after collecting the data, the author carried out image cleaning and preprocessing. The so-called image cleaning is to manually select the images after the images are collected, but the selection is not large, just remove the pictures that are not related to business English teaching. The so-called image preprocessing refers to the operation of feature extraction, segmentation, and preservation of the image, and the main purpose is to remove the information irrelevant to the research in the image and retain the information needed in the research. In this study, the author only did some research on the teacher's body shape in the whole classroom environment and did not need to consider the whole classroom environment and other factors, so the author extracted and processed the available information.

Using a large amount of sample data for training is an effective way for convolutional neural network models to improve the generalization ability. Usually, the total number of samples is insufficient or some types of samples are much lower than other types of samples, so it is necessary to artificially create “fake pictures” similar to the original pictures for training, which is also what we usually call data augmentation. There are two types of data enhancement. One is to directly enhance the data set. The newly enhanced data are saved in the original data set, which increases the number of samples in the data set, which is called offline data enhancement. The other is the enhancement performed during batch training. The enhanced data are only used for training, and the enhanced data are not put into the original data set. This method is called online enhancement.

This paper combines intelligent machine learning algorithms to improve the practical teaching mode of business English majors and proposes a practical teaching system for business English majors based on intelligent machine teaching to promote the effect of subsequent business English teaching.

## 2. Related Work

Reference [4] regards the interaction data of students in the intelligent teaching system as a kind of time series data and uses the hidden Markov model (HMM) to model the knowledge level of students. The model regards students' knowledge level as a latent variable, regards students' answering performance data as observation variables, and predicts their future performance according to their historical learning trajectory. At the same time, the model takes into account some accidental factors, namely the probability of a wrong answer and the probability of guessing right. However, the model does not consider the forgetting factor and believes that once students master a certain knowledge point, they will not forget, because the model believes that students have a short time to answer questions in the intelligent teaching system. Reference [5] considered forgetting factors in the knowledge tracking model, experimented on the KDDCup dataset, and concluded that considering forgetting can lead to better prediction performance. The early BKT model was not a personalized model, and it used all the student data to train a model. However, considering that different students have different knowledge levels and learning abilities, many scholars are committed to improving their personalization ability. Literature [6] proposes a personalized prior probability model PPS; that is, each student has his own initial probability. The author first trains a model with the data of all students and then adjusts its initial probability for each student, and the remaining probabilities are nonpersonalized parameters. Reference [6] used three heuristic methods to calculate personalized parameters: (1) random allocation; (2) set according to the answer of the first question; (3) directly set as the global correct rate of the student. The author uses it to predict the performance of students' last question, and the experimental results show that it is better than the traditional BKT model. Reference [7] trains a separate model for each skill of each student. Its shortcomings are also obvious. Due to the sparseness of the data, it cannot train a good model, and it will lead to the problem of too many parameters. Reference [8] does not use the EM algorithm to train the model, but uses a gradient search method, and can get personalized parameters during training. Reference [9] used hierarchical Bayesian models (HBM) to implement a personalized knowledge tracking model. Reference [10] analyzed the reasons for the low performance of traditional models and proposed the concept of model degradation; that is, when the parameters of the model have unreasonable values, the performance of the model will be seriously affected. For example, it is unreasonable for the probability of a wrong answer, and the probability of guessing to be higher than 0.5. The author's solution is to limit it to a reasonable range during training. The results improved the performance of the model. Reference [11] introduced the difficulty of the problem into the knowledge tracking model, which made the performance of the model substantially improved compared to the traditional BKT. Reference [12] proposed the Intervention-BKT model, which introduced the different guidance methods used by students each time they answered the question into the input node of the model, which improved the model structurally and improved its performance. Reference [13] uses Monte Carlo tree search (MCTS) to improve the prediction accuracy of the model. Since traditional models need a large amount of data support to perform well, data acquisition is usually very difficult, so they used reinforcement learning to generate some dummy data for model training, which greatly improved the model performance. Reference [14] adjusted the structure of the knowledge tracking model according to the characteristics of MOOC student data to predict the performance of MOOC students. Most of the personalized knowledge tracking models proposed by the previous experts are based on some heuristic methods, and the performance improvement of the models is not high. And they can only predict the performance of current students on the last few questions, but no new students, a problem known as the cold start problem. Furthermore, personalized knowledge tracking models increase the number of parameters and can lead to overfitting. Many studies have shown that clustering technology can effectively improve the performance of prediction models, and clustering technology is also one of the core technologies of intelligent teaching systems. Literature [15] proposed the concept of clustering knowledge tracking. The author used K-means and spectral clustering algorithm to cluster the student data into K clusters and then used the K groups of data to train K knowledge tracking models. The prediction results of multiple models are averaged as the final prediction result. Reference [16] used clustering algorithms in standardized test score prediction and used a logistic regression model for prediction.

## 3. Intelligent Classroom Image Digital Recognition

Business English is generally conducted through an online classroom teaching mode. Therefore, this paper studies the digital recognition algorithm of intelligent classroom image. In the digital recognition of business English, when the level of laser feedback increases gradually, the competition between modes will become increasingly fierce, resulting in the phenomenon of signal hysteresis, and the interference function *G*(*φ*_*F*_) changes with the initial phase *φ*_0_. As *φ*_0_ increases, when *G*(*φ*_*F*_) reaches point *φ*_0*B*_, it does not change along the path of the dotted line but jumps directly to point *φ*_0*B*_, resulting in a hysteresis phenomenon. Similarly, as *φ*_0_ decreases, when *G*(*φ*_*F*_) reaches *φ*_0*A*_, it will jump to point *φ*_0*A*_. In addition, when *φ*_0_=*φ*_0  *D*_, the interference function *G*(*φ*_*F*_) is zero. *φ*_*AB*_ refers to the phase change between *φ*_*AB*_ and *φ*_0*B*_, and *φ*_*A*  *D*_ refers to the phase change between *φ*_0*A*_ and *φ*_0  *D*_. The relationship between the phase change and the parameter *C* and *α* is given in the literature.

(1) $$\begin{array}{c} \varphi_{AB}=2\times \left[\sqrt{C^{2}-1}+\arccos\left(-1/C\right)-\pi \right], \end{array}$$

(2) $$\begin{array}{c} \varphi_{A\;D}=\frac{-\pi }{2}-\frac{C}{\sqrt{\alpha^{2}+1}}+\arccos\left(-\frac{1}{C}\right)+\arctan\left(\alpha \right)+\sqrt{\left(C^{2}-1\right)}. \end{array}$$

It can be known from formula (2) that the value of *α* can be obtained as long as the hysteresis width is obtained. A simple derivation process is given in the literature.

**Figure 1:**
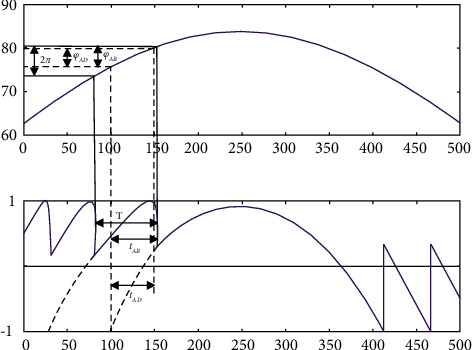
The relationship between time interval and phase interval when the object changes linearly.

As shown in Figure 1, the slopes of the transition points *φ*_0,*A*_, %*o*, *a* are infinite. Therefore, the derivative of *G* (%) at *Po*, *a*, *Po*, *R* is infinite.

(3) $$\begin{array}{c} \frac{dG\left(\varphi_{0}\right)}{d\varphi_{0}}=\frac{d\;\cos\left(\varphi_{0}\right)}{d\varphi_{0}}=-\sin\left(\varphi_{F}\right)\frac{d\varphi_{F}}{d\varphi_{0}}=\infty . \end{array}$$

According to the phase equation, we can get

(4) $$\begin{array}{c} \frac{d\varphi_{F}}{d\varphi_{0}}=\frac{1}{1+C\;\cos\left(\varphi_{F}+\arctan\left(\alpha \right)\right)}=\infty . \end{array}$$

Then, 1+*C*  cos(*φ*_*F*_+arctan(*α*))=0, and in addition, there are [17]

(5) $$\begin{array}{c} \varphi_{FA}=2\pi -\arccos\left(-\frac{1}{C}\right)-\arctan\left(\alpha \right), \end{array}$$

(6) $$\begin{array}{c} \varphi_{FB}=\arccos\left(-\frac{1}{C}\right)-\arctan\left(\alpha \right). \end{array}$$

Putting formulas (5) and (6) into phase equation (6), we can get

(7) $$\begin{array}{c} \varphi_{AB}=\varphi_{0,B}-\varphi_{0,A}=2\times \left[\sqrt{\left(C^{2}-1\right)}+\arccos\left(-\frac{1}{C}\right)-\pi \right],\\ \varphi_{0,B}=\arccos\left(-\frac{1}{C}\right)-\arctan\left(\alpha \right)+\sqrt{\left(C^{2}-1\right)},\\ \varphi_{0,A}=2\pi -\arccos\left(-\frac{1}{C}\right)-\arctan\left(\alpha \right)-\sqrt{\left(C^{2}-1\right)}. \end{array}$$

As shown in Figure 1, the value of *G*(*φ*_0_) is zero at the zero point *φ*_0,*D*_, so we have [18]

(8) $$\begin{array}{c} G\left(\varphi_{0}\right)=\cos\left(\varphi_{F}\right)=0. \end{array}$$

At this point, *φ*_*FD*_=(3/2)*π*.

Bringing it into the phase equation, we get

(9) $$\begin{array}{c} \varphi_{0,D}=\frac{3}{2}\pi -\frac{C}{\sqrt{\alpha^{2}+1}}. \end{array}$$

Therefore, $\varphi_{AB}=\varphi_{0,B}-\varphi_{0,A}=-\left(\pi /2\right)+\arccos\left(-\left(1/C\right)\right)+\arctan\left(\alpha \right)+\sqrt{\left(C^{2}-1\right)}$.

According to formulas (1) and (2), it can be known that the value of *α* can be calculated according to the values of *φ*_*AB*_ and *φ*_*AD*_. In the usual self-mixing interference signal, the time interval can replace the phase interval, and each integer fringe will correspond to the change of 2*π*, *φ*_*AB*_, and *φ*_*AD*_ can be expressed as [19]

(10) $$\begin{array}{c} \varphi_{AB}=\frac{2\pi t_{AB}}{T}, \end{array}$$

(11) $$\begin{array}{c} \varphi_{AD}=\frac{2\pi t_{AD}}{T}. \end{array}$$

Among them, *t*_*AB*_ and *t*_*AD*_ represent the time interval corresponding to *t*_*AD*_ and *φ*_*AD*_, and *T* is the time interval of the entire stripe.

As can be seen from the above figure, the calculation amount of each parameter is small. It can quickly get the result according to the formula, which obviously reflects the relationship between the time interval and the phase interval, and it can better understand the corresponding relationship. There is no interference in the simulation results, and each feature point can be clearly seen, and the numerical value is accurate to the thousandth, which makes the result more convincing.

**Figure 2:**
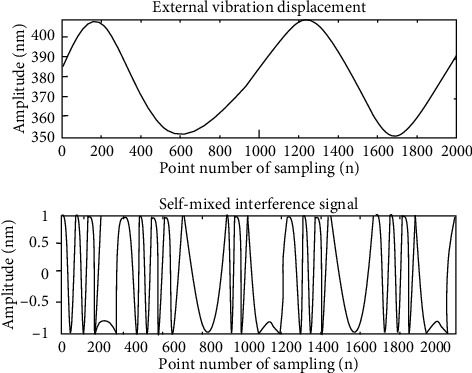
External vibration displacement and self-mixing interference signal.

However, in actual measurement, due to the irregularity of object motion, it is difficult to ensure that the back-and-forth linear motion is performed, which further increases the difficulty of measurement, so that the desired accuracy cannot be achieved. Therefore, this paper makes a sine wave motion to achieve modulation. We assume a sine wave periodic motion, and the resulting self-mixing interference signal is shown in Figure 2.

The steps of the symmetric folding algorithm are as follows:

1. The algorithm takes a period of self-mixing interference signal, including complete upper and lower fringes
2. The algorithm derives the self-mixing interference signal, the position of the positive and negative pulses is the position of the transition point, and the distance between adjacent pulses is *T*
3. The algorithm derives the sign function of the self-mixing interference signal, and the position of the negative pulse is the position of the zero point of the lower fringe
4. The algorithm finds the symmetrical point *t*_*R*_ and folds the self-mixing interference signal with respect to *t*_*R*_, obtains the time *t*_*AB*_ and *t*_*AD*_, and then obtains *t*_*AD*_ and *φ*_*AD*_ according to formulas (10) and (11)
5. The algorithm calculates the value of *α* according to formulas (1) and (2)

**Figure 3:**
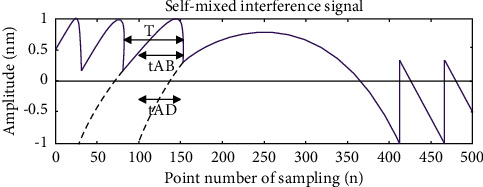
Self-mixing interference signal after symmetrical folding.

Based on the above basic principles, we use Matlab to simulate. First, a moderate feedback or strong feedback self-mixing interference signal is simulated, as shown in Figure 3, which shows that the symmetric folding algorithm given in this paper can be realized both in theory and simulation.

As can be seen from the above figure, the prepared numerical quantities can be obtained, and finally, the parameters can be obtained. However, it needs to be mentioned that the calculation above is based on the rightmost stripe. It can be seen from the figure that there are 3 stripes displayed, and the calculation results of each stripe are different. The accuracy of the calculation is also a very important point, and the factors affecting its accuracy mainly include the optical feedback level factor and the influence of different stripes. Every influencing factor is important, and an error will have a huge impact on the result.

### 3.1. Influence of Optical Feedback Level Factor on *α* Measurement

**Figure 4:**
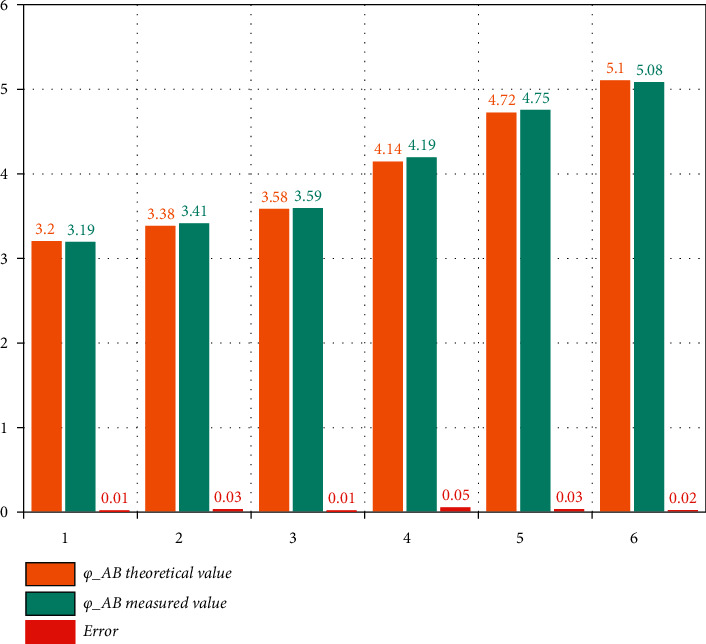
*α* is constant, measurement error of *φ*_*AB*_ theoretical value (left) and *φ*_*AB*_ measured value (right).

In order to more comprehensively verify the feasibility of the method, this paper analyzes the aspects of *C*, *α*, and stripe factors, respectively. When *α* is constant, the theoretical value of *φ*_*AB*_ can be obtained based on the given *C* according to formula (1), and then, *φ*_*AB*_ is obtained according to the simulated waveform. The simulation results are shown in Figure 4.

**Figure 5:**
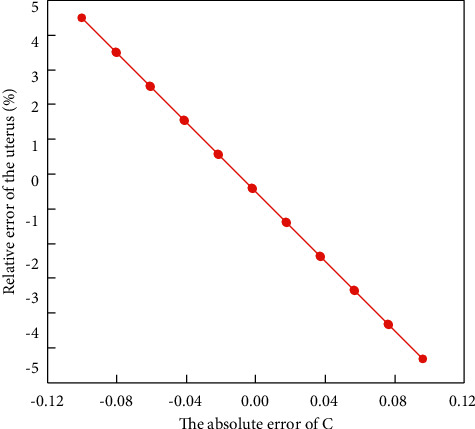
The effect of absolute error of *C* on *α*.

It can be seen from the above figure that there is an error between the measured value of *φ*_*AB*_ and the theoretical value, and the maximum can reach 5%. However, *φ*_*AB*_ and *φ*_*AD*_ directly affect the values of *C* and *α*. Because *φ*_*AB*_ directly affects the value of *C*, the measurement of *α* is also affected by *C*. In order to observe the influence intuitively, we assume that the standard value is *C* = 3, *a* = 2, the value of *φ*_*AD*_ is obtained, and then, the value of *φ*_*AD*_ is fixed, and the value of *C* is gradually increased from 2.9 to 3.1, and then, the value of *α* is obtained. The relationship between the two results is shown in Figure 5.

It can be clearly seen from the above figure that as the absolute error of *C* increases, the relative error of *α* also increases, and there is a linear relationship between them. It can be seen that the value of *C* obviously affects the calculation of *α*, so the accuracy of *C* must be ensured first. When the value of *C* is accurate enough, the value of *α* can be guaranteed to be accurate enough to make the calculation more accurate and convincing. Under the premise of accurately measuring *C*, measuring *α* is the most accurate. To sum up, the feedback level factor *C* value is the premise to ensure the accuracy of *α* measurement.

### 3.2. Influence of Stripes on *α* Measurement

**Figure 6:**
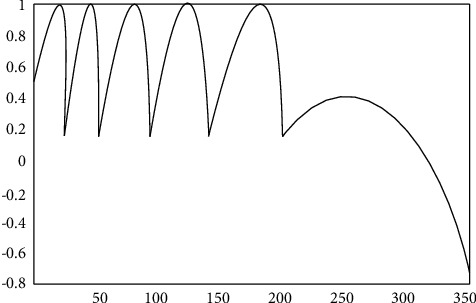
Self-mixing interference signal when the image light is moving sinusoidally.

In practical experiments, it is very difficult for us to achieve linear motion of image propagation. Therefore, in the experiment, only the PZT driver can be used to create, and in the simulation, the analysis of different stripes is obtained, and the calculated value is analyzed. The regular periodic motion is created by simulation, and the following self-mixing interference signal is obtained as shown in Figure 6. Since the movement is not linear, the spacing of each stripe shown is different.

**Figure 7:**
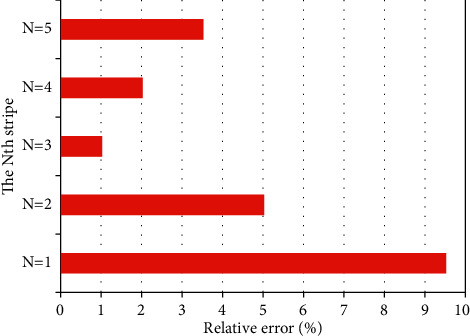
Relative errors corresponding to different stripes.

Through the above analysis and conclusions, different stripes are now used to calculate, and the results are not the same. The values of *C* and *α* were measured with the respective stripes. It is worth mentioning that in the measurement, the true value of *C* is used. In order to avoid errors, measurement values are not used, which is more accurate.  Figure 7 shows the relative errors of *α* measured by the above five fringes.

As can be seen from the above figure, the error measured by the third stripe in the middle is the smallest. This is because the middle fringe corresponds to the position of the equilibrium point, where the linear relationship between phase and time is better. Therefore, when calculating *C* and *α* later in this paper, it is more accurate to use the intermediate stripes for calculation. The simulation results show that the symmetric folding algorithm can achieve effective measurement of LEF under moderate feedback and strong feedback conditions.

Nowadays, in the measurement of phase, there are many methods, and the most used is the inverse trigonometric function. When using the inverse trigonometric function for calculation, a problem that will be involved is the phase unwrapping problem, which needs to be unwrapped to obtain the real phase. Since the self-mixing interference signal in the signal has characteristic points, such as transition points, the phase difference is 2*πC*/(1+*C*). When the value of *C* is large, the phase can basically be regarded as 2*π*. In this way, whenever a jump point is reached, adding or subtracting 2 Yuan can restore the desired phase step by step, and the obtained real phase can help us analyze the problem.

When calculated, the result will be outside the range of [−*π*, *π*]. In this case, the phase of the package will have multiple 2x jumps. On the mathematical basis, the process of phase wrapping can be represented by the following formula:

(12) $$\begin{array}{c} x_{w}\left(n\right)=w\left[x\left(n\right)\right], \end{array}$$

where *x*_*w*_(*n*) is the wrapped phase signal, *x*(*n*) is the original phase signal, and *w*[*·*] is the phase wrapping operator.

The four-quadrant function can be calculated by the following equation:

(13) $$\begin{array}{c} y\left(a,b\right)=\;\tan^{-1}\left(\frac{a}{b}\right)\;a>0,b>0\text{ first quadrant,} \end{array}$$

(14) $$\begin{array}{c} y\left(a,b\right)=\;\tan^{-1}\left(\frac{a}{b}\right)+\pi \;a>0,b<0\text{ second quadrant,} \end{array}$$

(15) $$\begin{array}{c} y\left(a,b\right)=\;\tan^{-1}\left(\frac{a}{b}\right)-\pi \;a<0,b<0\text{ third quadrant,} \end{array}$$

(16) $$\begin{array}{c} y\left(a,b\right)=\;\tan^{-1}\left(\frac{a}{b}\right)\;a<0,b>0\text{ fourth quadrant.} \end{array}$$

In the formula, *a* and *b* are real numbers.

The theoretical explanation is somewhat vague. In order to understand it better and more directly, we can first study it through simulation and use it to simulate the process of its unfolding. We assume that the signal is *x*(*n*)=6  sin(2*πft*), and according to formulas (13)–(16), the obtained wrapping signal is shown in Figure 7.

**Figure 8:**
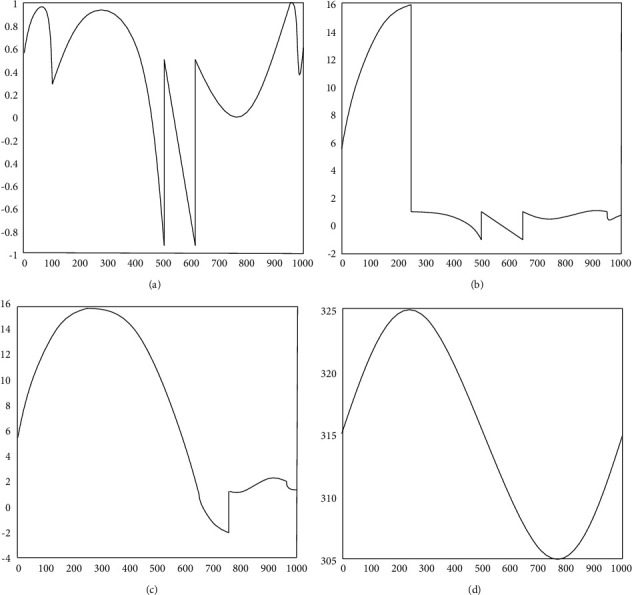
Simulation phase unwrapping process.

By observing Figures 8(a) and 8(b), it can be seen that the wrapping phase has a specific transition point, which cannot effectively and truly reflect the real information carried by the signal. At this moment, phase unwrapping is needed to turn it into a continuous self-mixing interference signal. This process is named phase unwrapping, and the basic principle of its unwrapping has been described above, and the following is its mathematical model and its principle.

The one-dimensional phase unwrapping formula can be expressed as follows:

(17) $$\begin{array}{c} w\left[\phi \left(i\right)\right]=\varphi \left(i\right)=\phi \left(i\right)+2\pi k\left(i\right), \end{array}$$

where *ϕ* is the wrapped phase, *φ* is the original phase, and k(i) is a sequence of integers.

The difference operator △ can be defined as

(18) $$\begin{array}{c} \Delta \left[\phi \left(i\right)\right]=\phi \left(i+1\right)-\phi \left(i\right), \end{array}$$

(19) $$\begin{array}{c} \Delta \left[k\left(i\right)\right]=k\left(i+1\right)-k\left(i\right). \end{array}$$

Combining formulas (18) and (19), we can get

(20) $$\begin{array}{c} \Delta \left\{w\left[\phi \left(i\right)\right]\right\}=\Delta \left[\phi \left(i\right)\right]+2\pi \Delta \left[k_{1}\left(i\right)\right]. \end{array}$$

Using the wrapping operator *w*[*·*] again for equation(20), we have

(21) $$\begin{array}{c} w\left\{\Delta \left\{w\left[\phi \left(i\right)\right]\right\}\right\}=w\left\{\Delta \left\{w\left[\phi \left(i\right)\right]\right\}\right\}=\Delta \left[\phi \left(i\right)\right]+2\pi \Delta \left\{\Delta \left[k_{1}\left(i\right)\right]+k_{2}\left(i\right)\right\}, \end{array}$$

where *k*_1_(*i*) and *k*_2_(*i*), respectively, represent the integer sequence of the two wrapping operators.

The package interval generated by *w*[*·*] is [−*π*, *π*], so Δ[*ϕ*(*i*)] is required to satisfy the condition −*π* < Δ[*ϕ*(*i*)] < *π*. Then, the subterm 2*π*Δ{Δ[*k*_1_(*i*)]+*k*_2_(*i*)} of formula (21) must be zero, and we get

(22) $$\begin{array}{c} \Delta \left\{\phi \left(i\right)\right\}=w\left\{\Delta \left\{w\left[\phi \left(i\right)\right]\right\}\right\}=w\left\{\Delta \left\{w\left[\phi \left(i\right)\right]\right\}\right\}. \end{array}$$

We assume that *ϕ*(0) is known, the phase unwrapping formula at any point *n* is as follows:

(23) $$\begin{array}{c} \phi \left(n\right)=\phi \left(0\right)+\sum_{i=1}^{n}w\left\{\Delta \left\{w\left[\phi \left(i\right)\right]\right\}\right\}=\phi \left(0\right)+\sum_{i=1}^{n}w\left\{\Delta \left[\phi \left(i\right)\right]\right\}. \end{array}$$

The wrapped phase A signal obtained in Figure 8 is processed according to the above steps. The expansion results are shown in Figures 8(b)–8(d). *B* unwrapped is the phase before the first jump point, *C* is the phase before the second jump point, and *D* is the fully unwrapped phase.

In order to apply the phase unwrapping method to recover the displacement of the external object, it is first necessary to understand the relationship between the self-mixing interference signal and the displacement of the external object. The displacement of the external object has a certain corresponding relationship with the optical phase, as shown in the following formula:

(24) $$\begin{array}{c} \left\{\begin{array}{c} x_{0}\left(t\right)=w_{0}\left(t\right)=2\pi \times \frac{L}{\left(\lambda_{0}/2\right)}=2\pi v_{0}\left(t\right),\\ x_{F}\left(t\right)=w_{F}\left(t\right)=2\pi \times \frac{L}{\left(\lambda_{F}/2\right)}=2\pi v_{F}\left(t\right). \end{array}\right. \end{array}$$

According to formula (1) mentioned above, the displacement and phase of the object can be obtained. Since *x*_0_(*t*) and *L* have a linear relationship, the change in *x*_0_(*t*) can be used to represent the change in L. According to the phase equation, *x*_0_(*t*) is obtained from *x*_*F*_(*t*). In summary, the key to the reconstruction of the displacement signal is to obtain the phase *x*_*F*_(*t*) when the optical feedback is included.

Since there is a known correspondence between the self-mixing interference signal and the phase containing the optical feedback, *x*_*F*_′(*t*) can be represented by *G*(*x*_*F*_(*t*)):

(25) $$\begin{array}{c} x_{F}^{\prime }\left(t\right)=\arccos\left[G\left(x_{F}\left(t\right)\right)\right]. \end{array}$$

It can be known from equation (25) that the wrapping phase *x*_*F*_′(*t*) can be obtained by taking the inverse cosine function of the self-mixing interference signal.

The wrapping phase *F*(*n*) is calculated by the arc cosine using the above method combined with the method in the literature, and the self-mixing interference signal is *G* (*n*), as shown in the following equation:

(26) $$\begin{array}{c} F\left(n\right)=\text{arccos}\left[G\left(n\right)\right]. \end{array}$$

Then, according to the principle of ±2*π*, the real phase *x*_*F*_(*n*) is obtained as follows:

(27) $$\begin{array}{c} x_{F}\left(n\right)={\left(-1\right)}^{n}\times \;\arccos\left[G\left(n\right)\right]+2\pi m. \end{array}$$

Finally, the final phase *H*(*n*) is calculated by equation (27):

(28) $$\begin{array}{c} H\left(n\right)=x_{F}\left(n\right)+C\;\sin\left[x_{F}\left(n\right)+\arctan\left(\alpha \right)\right]. \end{array}$$

According to the relationship between the displacement and the phase, the displacement *L*(*n*) can be obtained, as shown in the following formula:

(29) $$\begin{array}{c} L\left(n\right)=\frac{H\left(n\right)\lambda_{0}}{4\pi }. \end{array}$$

Finally, the displacement *L*(*n*) is obtained.

**Figure 9:**
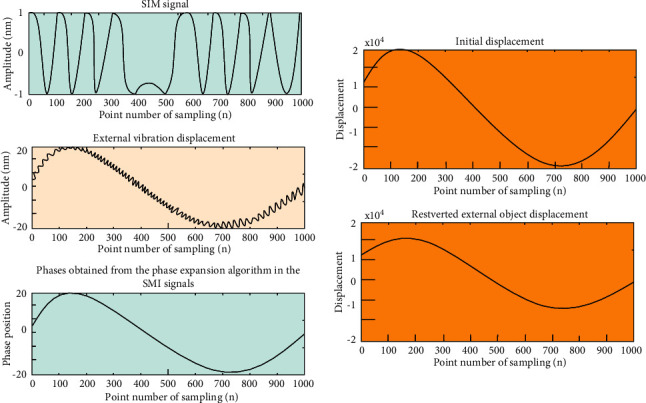
Object displacement of SMI signal unwrapping phase recovery when *C* = 0.8.

The simulation parameters are as follows: *f*_*s*_=2000*Hz*, *a*_1_=7*π*, *a*_2_=120*π*, *n* = 1000, and *α*=2. Under the condition that other parameters are kept unchanged, the specific changes of the signal can be observed by changing the value of *C*. First, under the condition of weak feedback, when *C* = 0.8, the simulation results are shown in Figure 9.

**Figure 10:**
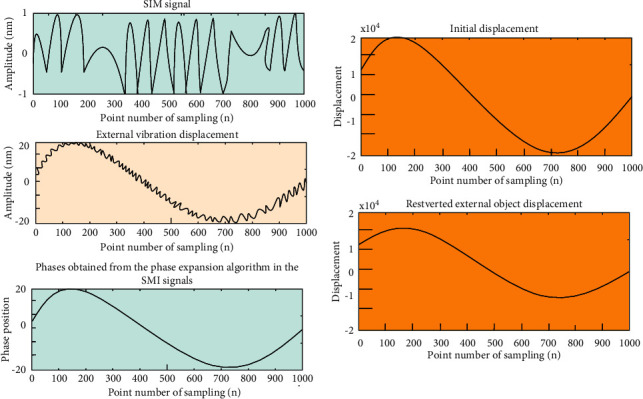
Object position of SMI signal unwrapping phase recovery when *C* = 3.

Under moderate feedback conditions (*C* = 3), the simulation results are shown in Figure 10, giving the initial displacement and the recovered external displacement.

**Figure 11:**
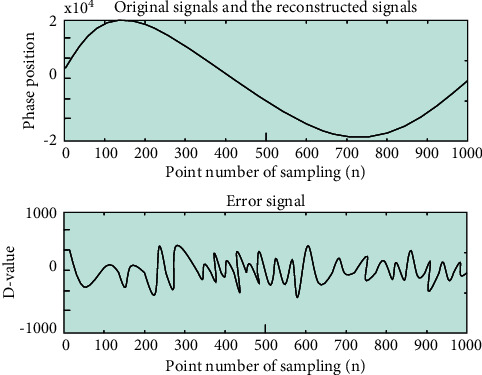
Error signal.

In the actual experiment, there will be a lot of noise interference that will affect the experimental results. To address this issue, it needs to be researched. In order to more carefully verify the feasibility of this method, we add noise to the original simulated signal, denoise it, and perform further displacement reconstruction. In the simulation, a noise signal of 20 dB is added on the basis of *f*_*s*_=2000*Hz*, *a*_1_=7*π*, *a*_2_=120*π*, *n* = 1000, *C* = 3, *α*=2. EEMD is used for denoising, C and *α* are calculated after denoising, and finally, reconstruction is performed, and the reconstructed result is compared with the original signal. As shown in Figure 11, the error signals of the two are shown.

**Figure 12:**
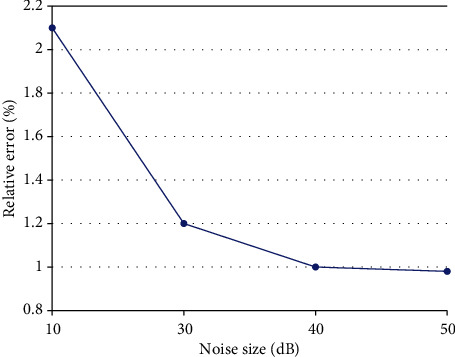
Relative error of each noise size.

As can be seen in Figure 11, the error still exists, the fitting degree of the two curves is almost similar, and the error is visible. However, the error signal graph can clearly show the magnitude of the error. Within the maximum acceptable absolute error, we present a plot of the relative error reconstructed by adding each SNR noise, as shown in Figure 12.

## 4. Verification of Practical Teaching Effect of Business English Major Based on Intelligent Machine Teaching

**Figure 13:**
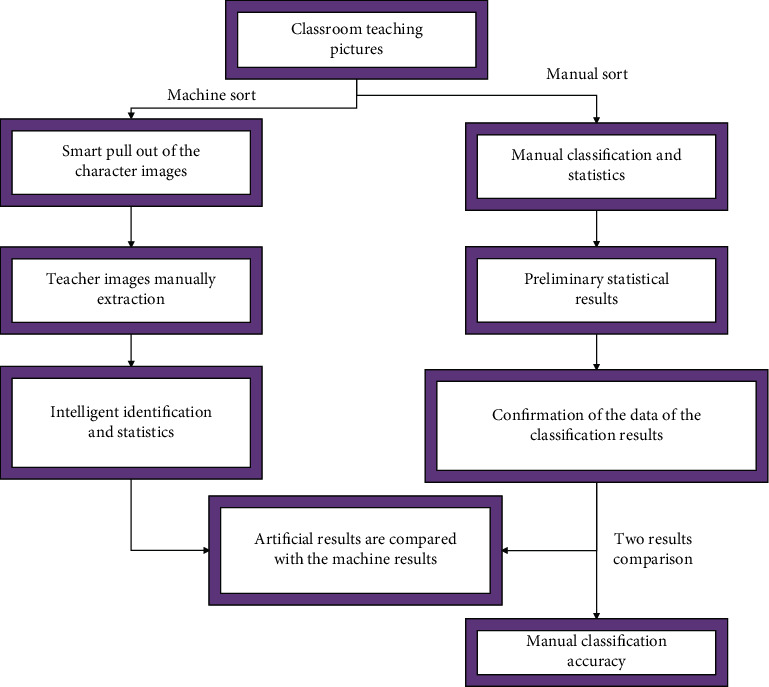
The process of practical teaching effect of business English major based on intelligent machine teaching.

In manual classification, we first select the data for preliminary classification statistics, then conduct another audit and statistics on the classified images, and use the audited data as the final reference data. In machine recognition, we first intelligently identify and cut out the character image and then extract the teacher's character image with a small amount of manual work. Finally, we carry out intelligent identification and statistics and compare with the final artificial statistics to obtain the accuracy of machine identification. The entire experimental process is shown in Figure 13.

The above intelligent teaching model is verified, and the teaching effect of the practical teaching system for business English majors based on intelligent machine teaching is counted, and the results shown in Table 1 are obtained.

Through the above research, we can see that the practical teaching system for business English majors based on intelligent machine teaching proposed in this paper has good effects and can play an important role in business English teaching.

## 5. Conclusion

The intelligent business English teaching system is a computer-aided business English teaching tool, and its research is an interdisciplinary subject involving many sciences. The goal of the intelligent business English teaching system is to establish an adaptive learning environment, provide students with personalized learning content, and improve students' learning benefits while reducing the burden of business English teaching for teachers. Intelligent business English teaching systems not only appear in traditional classrooms but also exist in large numbers on today's Internet platforms. Thanks to the development of science and technology, the audience of education has become wider and wider. Internet education has improved the shortcomings of traditional education, such as low fairness, low efficiency, and learning pain. Moreover, a large number of artificial intelligence-based business English teaching platforms have also appeared around us. The research shows that the practical teaching system for business English majors based on intelligent machine teaching proposed in this paper has good effects and can play an important role in business English teaching.

## Figures and Tables

**Table 1:** Statistics of practical teaching effect of business English major based on intelligent machine teaching.

Number	Teaching effect	Number	Teaching effect	Number	Teaching effect
1	88.88	23	93.88	45	88.97
2	91.95	24	91.51	46	92.22
3	93.57	25	88.67	47	93.79
4	93.21	26	89.53	48	92.63
5	92.92	27	92.43	49	89.88
6	93.69	28	88.09	50	89.93
7	91.54	29	88.87	51	91.53
8	91.62	30	88.23	52	89.03
9	88.23	31	90.12	53	93.31
10	92.40	32	93.21	54	89.95
11	91.77	33	89.48	55	91.07
12	92.15	34	89.69	56	92.61
13	93.32	35	92.31	57	88.95
14	92.53	36	90.06	58	93.11
15	89.91	37	88.26	59	90.59
16	93.67	38	93.19	60	88.70
17	91.18	39	89.81	61	92.61
18	89.77	40	90.10	62	93.49
19	92.50	41	90.09	63	88.35
20	90.14	42	91.33	64	92.43
21	90.02	43	92.05	65	92.97
22	88.96	44	92.08	66	88.85

## Data Availability

The labeled dataset used to support the findings of this study is available from the corresponding author upon request.
